# Effect of Marjoram Leaf Powder Addition on Nutritional, Rheological, Textural, Structural, and Sensorial Properties of Extruded Rice Noodles

**DOI:** 10.3390/foods12051099

**Published:** 2023-03-04

**Authors:** Siddharth Vishwakarma, Shubham Mandliya, Chandrakant Genu Dalbhagat, Jayshree Majumdar, Hari Niwas Mishra

**Affiliations:** Agricultural and Food Engineering Department, Indian Institute of Technology Kharagpur, Kharagpur 721302, India

**Keywords:** food-to-food fortification, natural fortificant, fortified rice noodles, marjoram

## Abstract

Food-to-food fortification is an emerging technique to enrich the micronutrients in foods. Pertaining to this technique, noodles could also be fortified with natural fortificants. In this study, marjoram leaf powder (MLP) at a level of 2–10% was used as a natural fortificant to produce fortified rice noodles (FRNs) through an extrusion process. The MLP addition caused a significant increase in the iron, calcium, protein, and fiber in the FRNs. The noodles had a lower whiteness index than unfortified noodles but had a similar water absorption index. The water solubility index increased significantly due to the higher water retention ability of MLP. A rheological study showed a minimal effect of fortification on the gelling strength of the FRNs at lower levels. The microstructural studies found incremental cracks, which facilitated a lower cooking time and hardness but had an insignificant effect on the cooked noodle texture. Fortification improved the total phenolic content, antioxidant capacity, and total flavonoid content. However, no significant changes in bonds were observed, but a reduction in the noodles’ crystallinity could be seen. The sensory analysis of the noodles reflected a higher acceptability of the 2–4% MLP fortified samples compared to the others. Overall, the MLP addition improved the nutritional content, antioxidant activity, and the cooking time but slightly affected the rheological, textural, and color properties of the noodles.

## 1. Introduction

Micronutrient malnutrition occurs due to deficiencies in minerals and vitamins in the diet. An iron deficiency is one of the most prevalent micronutrient deficiencies, and it leads to a reduction in the hemoglobin in the body, resulting in a decreased amount of oxygen in the blood [1], which is known as anemia. According to the World Health Organization, 60.2% of children under the age of five and 36.5% of pregnant women worldwide are anemic [2]. The situation is more alarming in India, as 68.9% of children and 57.2% of women were anemic in 2020 [3]. It should be taken into consideration that food fortification could extensively help to prevent and cure iron deficiency anemia. Thus, fortifying noodles can address this issue, as noodles are consumed globally by people of all ages, especially children.

Generally, noodles are deficient in micronutrients and adversely affect the health of growing children [4]. These noodles can be nutritionally improved by fortifying them with micronutrients, which will aid in the prevention of micronutrient deficits. The emerging strategy, which can complement the current strategies in the fight against micronutrient deficiencies, is food-to-food fortification (FtFF) [5]. In this method, micronutrient-dense foods, known as natural fortificants (NFs), are incorporated into food formulations to increase their micronutrient quantity. The dried forms of herbs, such as spearmint, marjoram, basil, thyme, peppermint, etc., have an immense potential to be used as NFs [6]. However, in one of our earlier studies, marjoram showed better nutritional and physiochemical properties than basil and spearmint [7].

Most noodles are prepared using wheat flour, which contains phytic acid, which reduces nutritional absorption [8], and the presence of gluten can cause complications for celiac patients [9,10]. By considering fortification and gluten-free requirements, rice flour (RF) can be used as an alternative primary ingredient for noodles [11]. The recent studies by Bouasla et al. [12] have shown that the addition of legume flours at levels of 10–30% improves the nutritional properties of rice pasta, resulting in a low cooking loss and acceptable sensory scores. Similarly, Liu et al. [13] found that incorporating less than 5% black garlic pulp into rye flour noodles had no significant impact on their sensory properties. However, lower fortification levels of 2 and 3% were the most acceptable in terms of color, cooking quality, microstructure, and textural properties. Adding 5% anthocyanin-rich extracts from black soybean coats to wheat noodles had an insignificant effect on texture properties such as hardness, elasticity, cohesiveness, chewiness, and resilience. However, substituting 7.5% or 10% resulted in significant changes in the texture and the collapse of the gluten protein network structure. Szydłowska-Tutaj et al. [14] studied the development of mushroom-enriched pasta using four different varieties (lion’s mane, reishi, enoki, and maitake). All the varieties increased the nutritional content, particularly ash, protein, and dietary fiber. Based on the sensory and nutritional aspects, the recommended levels of supplementation in pasta were 5% for lion’s mane and reishi, 7.5% for enoki, and 10% for maitake mushrooms. Bhandari et al. [15] found that incorporating 2.5–10% herbs into pasta improved its functional properties, increased the quantity of phytochemical constituents with an elevated antioxidant potential, and altered the molecular and structural interactions. Fortification must increase the targeted nutritional component along with having negligible detrimental effects on other properties such as texture, sensory properties, etc. An attempt was made to use dried marjoram leaf powder (MLP) as an NF and to fortify rice noodles at different levels to see any changes in their physicochemical, nutritional, rheological, textural, and morphological properties.

## 2. Materials and Methods

### 2.1. Materials

The dried marjoram leaves were purchased from Neutraved Xpotim Enterprises (Indore, Madhya Pradesh, India). The broken rice (*Swarna Cv.*) was purchased from Golden India Food Park (Pollachi, Tamil Naidu, India). All ingredients were ground, passed through a 150 µm sieve, and packed in ziplock pouches.

### 2.2. Preparation of Fortified Rice Noodles

The marjoram powder was mixed into RF at five levels, using 100% RF as a control for developing fortified rice noodles (FRNs). Five fortification levels (FL) were developed, which were F0 (MLP:RF—0:100 *w/w*), F2 (MLP:RF—2:98 *w/w*), F4 (MLP:RF—4:96 *w/w*), F6 (MLP:RF—6:94 *w/w*), F8 (MLP:RF—8:92 *w/w*), and F10 (MLP:RF—10:90 *w/w*). The samples were put in the planetary mixer (M/s Reico Equipment & Instrument, Kolkata, India) for blending and moisturizing up to 32.5% wet basis (wb) followed by overnight tempering in a refrigerator. Samples were extruded to develop FRNs using KETSE 20/40 co-rotating twin-screw extruder (Brabender GmbH, Germany). Based on the preliminary experimental trials, the extrusion process condition was set to have 110 °C die temperature, 80 rpm screw speed, and 13 rpm feeder speed. FRN samples were dried in a recirculating hot air tray dryer (Basic Technology Pvt. Ltd., Kolkata, India) at 40 °C and 1 m/s air speed for 4 h. After that, the samples were placed in ziplock bags and stored at room temperature (25 °C). A few of the dried noodles were ground, sieved (150 µm), and stored in ziplock bags. All the formulations were subjected to the same process treatment.

### 2.3. Physicochemical Properties

#### 2.3.1. Proximate Analysis

Moisture (method 930.15), ash (method 923.03), crude fat (method 920.39), crude fiber (method 978.10), and crude protein (method 978.04) of samples were measured in triplicate using the standard analytical process of the AOAC [16], and carbohydrate was evaluated by difference. The mineral compositions were examined with an atomic absorption spectrometer (Model: AAnalyst 700, PerkinElmer, USA) as described by Yadav et al. [17].

#### 2.3.2. Color Characteristics

The color of the FRN samples was analyzed using a colorimeter (CM-5, Konica Minolta, Tokyo, Japan) following the method described by Dalbhagat and Mishra [18]. The analysis was conducted under a D65 illuminant with a 10° observer angle, and the color was represented in Hunter lab coordinates, including lightness (L*), redness (a*), and yellowness (b*). The L* value ranges from 0 (black) to 100 (white); a* values indicate redness (+a) or greenness (−a), and b* values indicate yellowness (+b) or blueness (−b). The colorimeter was calibrated using black and white specimens. For measurement, a 35 mm diameter dish was filled with powdered FRN samples with a particle size range of 150–250 µm, and triplicate readings were taken to calculate the average value. Equations (1) and (2) were used to determine the overall color difference (∆E) and whiteness index (WI), respectively, as described by Mandliya et al. [19] and Dalbhagat and Mishra [20]:

(1) $$\Delta E=\sqrt{{\left(L-L^{*}\right)}^{2}+{\left(a-a^{*}\right)}^{2}+{\left(b-b^{*}\right)}^{2}}$$

(2) $$WI=100-\sqrt{{\left(100-L^{\prime }\right)}^{2}+{a^{\prime }}^{2}+{b^{\prime }}^{2}}$$

where L*, a*, and b* are the color parameters of F0 and where L, a, and b are the color parameters of FRNs. L′, a′, and b′ are the color parameters of all noodles.

#### 2.3.3. Water Absorption Index (WAI) and Water Solubility Index (WSI)

FRN powder (2 g) was vortexed with distilled water (20 mL) for 15 min and was then centrifuged at 4000 rpm for 20 min. The supernatant was collected in a preweighed Petri plate and was oven-dried at 95 °C overnight. The precipitate was collected in the preweighed centrifuge tubes. The WAI and WSI were assessed using Equations (3) and (4), respectively [11].

(3) $$\mathrm{WAI}\;\left(g/g\right)=\frac{\mathrm{Weight}\;\mathrm{of}\;\mathrm{gel}\;\left(g\right)}{\mathrm{Weight}\;\mathrm{of}\;\mathrm{sample}\;\mathrm{flour}\;\left(g\right)}$$

(4) $$\mathrm{WSI}\;\left(\%\right)=\frac{\mathrm{Weight}\;\mathrm{of}\;\mathrm{dried}\;\mathrm{supernatant}\;\left(g\right)}{\mathrm{Weight}\;\mathrm{of}\;\mathrm{sample}\;\mathrm{flour}\;\left(g\right)}\times 100$$

### 2.4. Rheological Properties

#### 2.4.1. Pasting Properties

The pasting profile of FRN samples was evaluated using a rheometer (MCR 52 Rheometer, Anton Paar, Graz, Austria) based on Dalbhagat and Mishra [20] method. The canister of the instrument was loaded with the 10% (*w/v*) flour suspension. The temperature profile of pasting analysis was set at 50 °C with incubation for 1 min, heating was set at 95 °C at 12 °C/min rate, holding was set at 95 °C for 2.5 min, cooling was set at 50 °C at 12 °C/min rate, and holding was set at 50 °C for 2 min. Each sample’s pasting curve was used to calculate its peak viscosity (PV), peak temperature (PT), holding strength (HS), breakdown viscosity (BV), final viscosity (FV), setback from peak viscosity (SPV), and setback from trough viscosity (STV). The gel formed from the analysis was kept in the Petri dish inside the refrigerator at 4 °C overnight. This gel was further used for dynamic, creep, and steady shear analyses (SSA). 

#### 2.4.2. Dynamic Oscillatory Test

Amplitude sweep test (AST) of gel was conducted in the same rheometer at 25 °C using the parallel plate geometry of 25 mm diameter with a gap of 2 mm. The strain was varied from 0.1 to 100%, and the frequency (ω) was kept at 5 rad/s. Storage modulus (G′) and loss modulus (G″) were determined as a function of strain to identify the linear viscoelastic regime of all fortified gels [21]. A frequency sweep test (FST) was conducted at 25 °C during which the ω ranged from 1 to 100 rad/s, and the strain was kept within the identified linear viscoelastic regime. The G′ (Pa) and G″ (Pa) were determined as a function of the ω [21]. 

#### 2.4.3. Steady Shear Analysis (SSA)

An SSA of gels was carried out using the same rheometer and configuration. The viscosity of gels was measured against 0.0001 to 1 s^−1^ shear rate (SR) at 25 °C [21].

#### 2.4.4. Creep Recovery Test

Using the same rheometer, a creep test was performed on the gel. A fixed shear stress of 300 s in the linear viscoelastic range was given to the gel sample at 25 °C. The sample was then rested for 600 s during the recovery period in order to regain the elasticity of the deformation [22]. 

### 2.5. Microstructure

Scanning electron microscopy (ZEISS EVO, Göttingen, Germany) at a voltage of 5 kV was used to examine the microstructure of the FRN samples, RF, and MLP. The samples were mounted on studs and were coated with a layer of gold palladium (360 Å thick) under a vacuum followed by scanning electron microscopy [23]. The images were captured at magnifications of 100×, 500×, and 1000×.

### 2.6. Cooking Characteristics

#### 2.6.1. Cooking Time

The cooking time (CT) of FRN samples was measured based on the method of Bhandari et al. [15]. A total of 5 g of FRN samples was added to the boiling water. A tiny strand of FRN samples was taken out and pushed between glass slides at 1 min intervals to check for the presence of a white core. When the white core disappeared, the FRN cooking came to an end.

#### 2.6.2. Solid Loss and Water Absorption Ratio

The water absorption ratio (WAR) and solid loss (SL) during cooking were estimated according to Dalbhagat and Mishra [11]. A total of 5 g of FRN samples was added and cooked in boiling distilled water. At the end of cooking, the leached solids were collected in the glass Petri dish and were dried overnight at 105 °C. Simultaneously, the weight of cooked FRNs was taken accurately, and WAR was calculated. SL was determined using the weight of dried leached solids.

### 2.7. Textural Analysis

The hardness of uncooked FRNs was determined using a texture analyzer (CT3, Brookfield Technologies Corporation, 50 kg load cell). To perform this test, a single noodle strand (thickness: 2.00 ± 0.01 mm) was located longitudinally upon the fixture base table (TA-BT-KIT), ensuring it was centrally aligned under the 60 mm wide knife edge TA7 probe. Test type, target type, target value, hold time, trigger load, test speed, post-test speed, and cycle count were set to compression, distance, 1 mm, 0 s, 0.1 N, 1.00 mm/s, 1.00 mm/s, and 1, respectively. The highest force needed to break the noodles was used to determine the hardness. All the measurements were done in ten replicates [24]. Using the same texture analyzer with a 25.4 mm cylindrical TA11/1000 probe, three cooked FRN strands with length of 20 mm were placed longitudinally upon the same fixture base table, ensuring the coverage of the sample by the probe during the textural profile analysis (TPA). Test type, target type, target value, hold time, trigger load, test speed, post-test speed, and cycle count were set to TPA, distance, 1 mm, 0 s, 0.1 N, 1.00 mm/s, 1.00 mm/s, and 2, respectively. The TPA was measured in terms of hardness, adhesiveness, gumminess, springiness, chewiness, and cohesiveness as described by Nithya et al. [25]. All the measurements were done five times.

### 2.8. Total Phenolic Content (TPC), Antioxidant (AOX) Capacity, and Total Flavonoid Content (TFC)

The TPC of the samples was estimated by using the method described by Mandliya et al. [26] with slight modifications. A 1 g sample was mixed with 20 mL of water and was then subjected to ultrasound treatment in a water bath for 30 min at 35 °C. The mixture was then vortexed for 1.5 min before being centrifuged at 10,000 rpm for 10 min at 20 °C. The resulting supernatant was collected as an extract for TPC and TFC analysis. For TPC analysis, 0.25 mL of the extract was mixed with 0.5 mL of Folin–Ciocalteu reagent (diluted 10 times) and 0.75 mL of 20% *w/v* sodium carbonate and was then brought up to 4 mL using Millipore water. The mixture was incubated in the dark for 90 min, and the intensity of the developed blue color was measured by taking absorbance at 760 nm using a UV–visible spectrophotometer (UV-1800, Shimadzu Scientific Instruments Inc., Kyoto, Japan). Gallic acid was used as a standard for total phenolics, and TPC was expressed in “mg of Gallic Acid Equivalents” or mg GAE per gram of dry weight.

The AOX capacity of the samples was determined in terms of 1,1-diphenyl-2-picrylhydrazyl (DPPH) radical scavenging capacity following the procedure outlined by Dubey et al. [27]. Sample extract (0.2 mL) prepared using ethanol as solvent was mixed with DPPH solution (0.95 mL) and was brought up to 4 mL using ethanol. The mixture was then kept in the dark for 30 min before measuring the absorbance at 517 nm. The AOX capacity of the extract was expressed as gallic acid equivalent in terms of mg GAE per gram of dry weight using the formula presented below in Equation (5).

(5) $$A\mathrm{OX}\;(\mathrm{mg}\;\mathrm{GAE}/g)=\frac{\Delta \mathrm{Abs}\;\mathrm{of}\;\mathrm{sample}}{\Delta \mathrm{Abs}\;\mathrm{of}\;\mathrm{GA}}\times \frac{V}{W}\times C_{\mathrm{GA}}$$

where mg GAE refers to milligrams of gallic acid equivalent AOX capacity, ΔAbs of the sample indicates the change in absorbance after adding the sample, ΔAbs of GA refers to the change in absorbance obtained from the standard calibration curve when the same volume of the GA standard solution as that of the sample was added, CGA represents the concentration of the GA standard solution, V stands for the volume of the centrifuged extract, and W represents the weight of the sample.

The TFC was determined using the method described by Bouajila et al. [28], which is based on the formation of a complex between phenolic compounds and aluminum trichloride (AlCl_3_). The water-extracted sample (0.2 mL) was first diluted with Millipore water (2 mL) and was then mixed with sodium nitrite (0.15 mL, 5% *w/v*). After 5 min, AlCl_3_ (0.15 mL, 10% *w/v*) was added to the mixture, which was then allowed to stand for 6 min. Next, sodium hydroxide (1 mL of 1 M NaOH) was added to the solution, which was brought up to 5 mL using Millipore water and was incubated in the dark for 15 min. The TFC was measured using a UV-visible spectrophotometer at 510 nm, and the TFC was expressed in mg of quercetin equivalent (QE) per gram of dry weight (mg QE/g DW) using its calibration curve.

### 2.9. Fourier Transform Infrared (FTIR) Spectroscopy

FTIR spectroscopy was performed with an FTIR spectrophotometer (NICOLET 6700, Thermo Fisher Scientific, Waltham, MA, USA) with a frequency range of 400 to 4000 cm^−1^. Potassium bromide was mixed into the ground samples to create a pellet, which was then hydraulically compacted and exposed to an IR beam. The absorbance at 995, 1020, and 1047 cm^−1^ was checked, as they represent the crystalline structure of starches, and the absorbance ratio (1047/1022 and 1022/995) was calculated [29]. 

### 2.10. X-ray Diffraction Analysis

X-ray diffraction (XRD) patterns of FRN samples were analyzed using the method stated by Yadav et al. [29] with minor modifications. An X-ray diffractometer (XRD, D2 PHASER, M/s Bruker, Germany) was used for the analysis. The diffractogram was generated over a 5° to 40° diffraction angle (2θ) at a 2.68°/min stepping rate. Relative crystallinity (%) was determined using Equation (6).

(6) $$Relative\;crystallinity\;\left(\%\right)=\frac{\;Crystalline\;peak\;area}{Crystalline\;peak\;area+Amorphous\;area}\times 100$$

### 2.11. Sensory Analysis

The sensory evaluation was performed with an untrained panel of 25 people aged 20–40 yrs. On the basis of a nine-point hedonic analysis, the participants were asked to score each sample’s quality attributes (taste, color, aroma, texture, appearance, stickiness, and overall acceptability) on a scale from nine (liked extremely) to one (extremely disliked). In order to avoid prejudice, participants only received a limited amount of information on FRN. When required, research assistants provided assistance to panelists during the sensory assessment.

### 2.12. Statistical Analysis

All the experiments were conducted in triplicates. Tukey test was done for the de-termination of significant terms (*p* < 0.05) with Minitab 17.1.0 (Minitab LLC, USA). The FTIR data analysis, crystalline area, amorphous area under the X-ray spectral region, and principal component analysis (PCA) were done using Origin 18 (OriginLab Corporation, USA). Pearson correlation analysis was done using IBM SPSS Statistics 22.

## 3. Results and Discussion

### 3.1. Physicochemical Properties

#### 3.1.1. Proximate Composition and Mineral Analysis

The proximate and mineral composition of the FRNs is shown in Table 1. MLP was found to be highly rich in iron, calcium, protein, and fiber. The addition of MLP in the FRNs caused a significant increase (*p* < 0.05) in iron from 0.21 to 5.5 mg/100 g. A significant increase (*p* < 0.05) was noticed in calcium from 5.7 mg/100 g in F0 to 25.4 mg/100 g in the F10 sample. However, a significant (*p* < 0.05) but slight improvement in fiber was observed. The protein content was significantly (*p* < 0.05) increased with up to 4% MLP. The ash content significantly (*p* < 0.05) improved due to MLP, but its content was low. Similarly, a low fat content was observed with fortification. In the case of zinc, an insignificant (*p* > 0.05) increase in the FRNs was observed at each subsequent level. Similar higher nutrition values of marjoram were corroborated by Vishwakarma et al. [6]. The analysis showed that the FRNs contained high iron, calcium, protein, and fiber with a low fat content.

#### 3.1.2. Color

**Figure 1:**
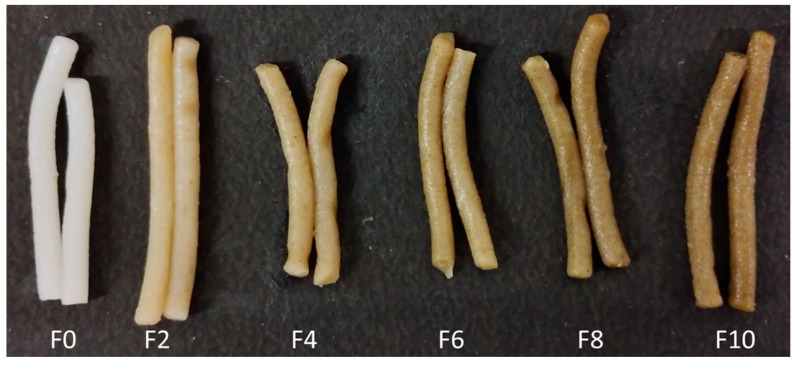
Cooked noodles based on F0 and FRN formulations added; F0 represents 0% MLP, F2 represents 2% MLP, F4 represents 4% MLP, F6 represents 6% MLP, F8 represents 8% MLP, and F10 represents 10% MLP.

The determination of the noodles’ color is important for determining its acceptability. The color attributes, i.e., L, a, b, WI, and ΔE of the formulations, are presented in Table 2, and the samples are in Figure 1. The WI of the RF was higher than those of F0 and all the formulated noodles, whereas the WI of MLP was lower than that of the RF, F0, and the other formulations. The ‘L’ value decreased significantly (*p* < 0.05) in the FRNs with an increase in the concentration of MLP, except for F10. This reduction in the ‘L’ values was due to the green color of MLP [8]. The color imparted due to MLP was prominently observed in the ‘a’ value, which increased significantly (*p* < 0.05) with fortification. Similar observations were noted for the ‘b’ values of the noodles. The MLP addition caused a significant (*p* < 0.05) increase in ΔE, which, in due course, decreased the WI, and this can be visually seen in Figure 1. Similar changes in the color properties of wheat pasta were noticed by Lisiecka et al. [30] when incorporating *Cistus incanus* L. leaves.

#### 3.1.3. Water Absorption Index (WAI) and Water Solubility Index (WSI)

The swelling and water-holding capacity of the samples could be observed from the WAI values, while the quantity of components that were solubilized in water could be presented as the WSI (Table 3) [8]. The RF exhibited the minimum WAI value, while MLP showed both the maximum WAI and WSI values. It can be inferred that a greater presence of absorbent and soluble components is in MLP compared to the other samples. However, the MLP addition exhibited the negligible effect of MLP on the rice starch matrix, which was an important component that caused water absorption and resulted in higher values of the WAI of F0. Similar results showing no significant changes in the WAI were corroborated by Bouasla et al. [12]. The WSI values were significantly (*p* < 0.05) increased due to the addition of MLP. However, no increase (*p* > 0.05) was noticed at higher MLP levels, i.e., at 8% and 10%. The higher WSI was probably due to the higher water retention ability of MLP. Results similar to those of the WSI were seen by Bhandari et al. [15] while incorporating therapeutic herbs into pasta. The WAI and WSI outcomes reflected the negligible starch conversion due to fortification.

### 3.2. Rheological Properties

#### 3.2.1. Pasting Properties

**Figure 2:**
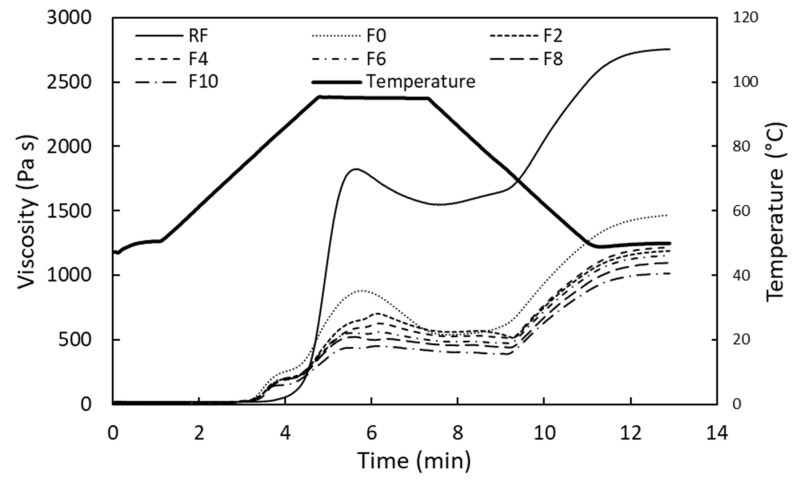
Pasting curve of RF, F0, and FRN samples. RF represents rice flour, F0 represents 0% MLP, F2 represents 2% MLP, F4 represents 4% MLP, F6 represents 6% MLP, F8 represents 8% MLP, and F10 represents 10% MLP.

The PV, PT, HS, BV, FV, SPV, and STV values define the pasting properties of the FRNs. The details of the pasting properties are shown in Table 4. A significant drop in the pasting curves (Figure 2) of all the FRNs was observed compared to the RF, which was due to starch gelatinization during extrusion cooking. Similar outcomes were presented by Dalbhagat et al. [11] for fortified rice kernels. The pasting properties of all the FRN samples were quite adjacent to each other (Figure 2), and their values decreased with an increase in the MLP addition (Table 4), except for PT and SPV at 10% MLP. The PT values had no significant difference (*p* > 0.05) with the substitution of MLP. There was no significant difference in the PV of F0 and F2; however, it significantly decreased after the 2% MLP addition. Similar observations were noted for HS and BV at MLP substitutions ≥ 6% (Table 4). Adding 2% MLP imparted significant (*p* < 0.05) changes in FV, SPV, and STV. There was no significant (*p* > 0.05) difference in the FV or STV values from 2% to 8% MLP. Similarly, the SPV values of F8 and F10 had no significant difference from F2. These results reflected the insignificant effect of MLP in starch gelatinization for up to the 4% level and a significant effect in the retrogradation properties for the 2% MLP addition. These outcomes are in accordance with the WSI results.

#### 3.2.2. Dynamic Rheological Properties

**Figure 3:**
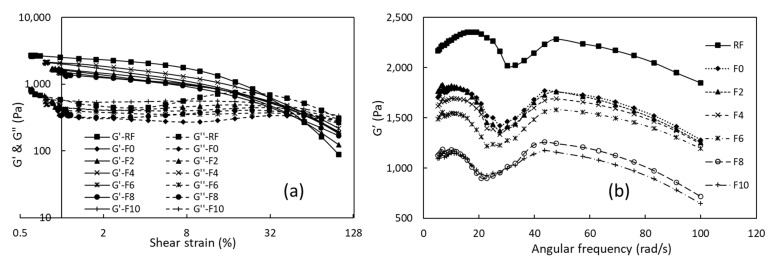
Dynamic rheology of gels of RF, F0, and FRN samples: (**a**) amplitude sweep test and (**b**) frequency sweep test. G′ and G″ are the storage and loss modulus, respectively. RF represents rice flour, F0 represents 0% MLP, F2 represents 2% MLP, F4 represents 4% MLP, F6 represents 6% MLP, F8 represents 8% MLP, and F10 represents 10% MLP.

The AST and FST were performed on the gel prepared from the pasting analysis. The AST (Figure 3a) showed a 5% linear viscoelastic region (LVER). Similarly, a 6–8% LVER was shown for brown and milled rice gels by Mariotti et al. [21]. The MLP caused a slight decrease in the G’ and G” of the FRNs.

The FST was performed within the LVER shown in Figure 3b. A higher G’ value was observed for the RF gels, which revealed its greater strength with the presence of high native starch. However, the extrusion caused a significant decrease in the gelling strength, and each FRN sample showed a similar profile. The MLP slightly decreased the G’ of the gels (for the 2 to 6% substitutions); in spite of this, the significant decrease was only observed at higher substitution levels (8–10%). Comparable outcomes were also noticed by Ma et al. [22] and Lui et al. [13].

#### 3.2.3. Creep and Steady Shear Analyses

**Figure 4:**
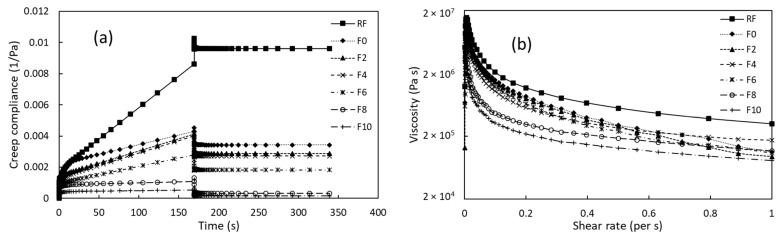
Rheological measurement of gels of RF, F0, and FRN samples: (**a**) creep recovery test and (**b**) steady shear analysis. RF represents rice flour, F0 represents 0% MLP, F2 represents 2% MLP, F4 represents 4% MLP, F6 represents 6% MLP, F8 represents 8% MLP, and F10 represents 10% MLP.

The creep test shows the degree of elastic deformation and the rate of viscous flow due to the application of small shear stress and the total recoverable elastic deformation after removing the stress. This test is generally used for determining the strength of doughs [31] and gels [32]. Figure 4a shows the creep behavior of the different gel samples at 500 Pa shear stress. The creep test showed a substantial decrease in creep compliance due to extrusion processing. Similar to the FST results, a trivial difference in the gelling strength between the F2 and F0 samples could be observed. Furthermore, a similar gelling strength could be seen among the F2 to F6 samples. However, a significant (*p* < 0.05) difference in creep compliance could be seen in the F6 and F8 samples, whereas there was a negligible difference (*p* > 0.05) between the F8 and F10 samples. Similar creep behavior of cereal starch gels was seen by Feng et al. [33].

The SSA was performed to check the flow behavior of the gels, and the results are shown in Figure 4b. All the gels exhibited pseudoplastic behavior, i.e., the viscosity decreased with an increase in SR. Attributed to the lower gelling strength of all the gels, the maximum SR was kept as low as 1 s^−1^. The gel viscosity of the RF was higher than that of the extruded samples, whereas a slight decrease in viscosity with the MLP addition was observed. On the other hand, at higher levels (F8 and F10), a considerable decrease in viscosity was observed as compared to others. Results similar to those of the SSA study were observed by Feng et al. [33]. All the rheological measurements of the RF and the FRNs indicated an insignificant effect of MLP at lower levels and a superior effect at higher levels. 

### 3.3. Microstructure

**Figure 5:**
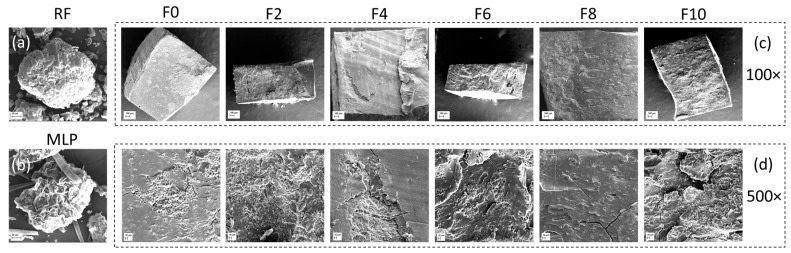
Microstructure of (**a**) RF at 1000×, (**b**) MLP at 1000×, (**c**) FRNs at 100×, and (**d**) FRNs at 500×. RF represents rice flour, MLP represents marjoram leaf powder, F0 represents 0% MLP, F2 represents 2% MLP, F4 represents 4% MLP, F6 represents 6% MLP, F8 represents 8% MLP, and F10 represents 10% MLP.

The microstructures of the RF, MLP, and the FRNs at different magnifications are shown in Figure 5. The RF contained the maximum amount of native starches, and MLP majorly contained fiber, protein, and minerals as indicated by the nutritional analysis. The longitudinal and transverse sections of the F0 samples contained very few cracks. However, with the MLP addition, the number of small cracks significantly increased, which could be seen at 100× and 500× magnifications. The proliferation of the roughness of the surface could be observed at higher FLs. These minor cracks may affect the strength of the noodles but facilitated easy water penetration during cooking. A similar increase in the number of cracks was sighted by Bhandari et al. [15] in their morphological study of pasta fortified with herbs.

### 3.4. Cooking Characteristics

**Figure 6:**
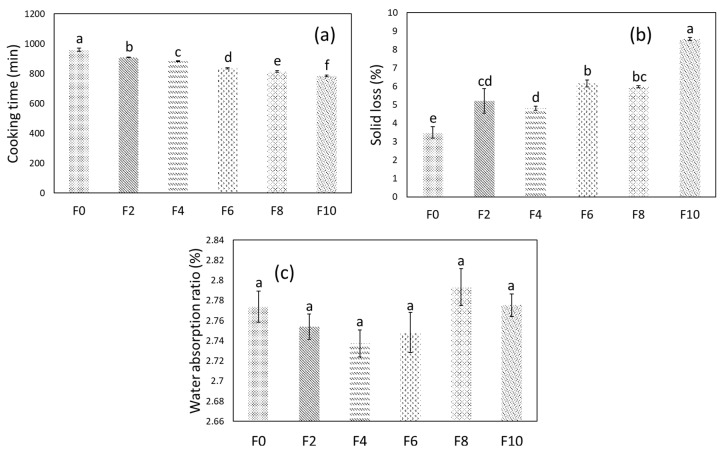
Cooking characteristics of F0 and FRNs: (**a**) CT, (**b**) SL, and (**c**) WAR. Different letters on the top of the bar show significant differences (*p* < 0.05). F0 represents 0% MLP, F2 represents 2% MLP, F4 represents 4% MLP, F6 represents 6% MLP, F8 represents 8% MLP, and F10 represents 10% MLP.

The CT, SL, and WAR of the FRNs are shown in Figure 6. The CT of the noodles decreased significantly (*p* < 0.05) with MLP from 16 min to 13 min and 5 s (Figure 6a), whereas SL followed the opposite trend (Figure 6b). However, no significant change (*p* > 0.05) was observed in the WAR. The decrease in CT was probably due to an increase in the minor cracks as shown by the microstructure analysis. Furthermore, the pasting studies also showed a decrease in the starch that was available for gelatinization, which can facilitate faster cooking. On the other hand, a higher SL was attributed to the lower gelling strength of the noodles with MLP substitution. However, the SL of all the noodle samples was below 9%, and similar SL values were found by Rathod and Annapure [34] when developing lentil-based noodles through extrusion. Furthermore, fortification had a negligible impact on the absorption property (Figure 6c) of the noodles as was seen earlier with the WAI results. The MLP addition resulted in better cooking properties of the noodles with a decrease in CT and the acceptable SL and WAR values. Similar results were documented by Bhandari et al. [15], representing a low CT and increased SL due to herb addition.

### 3.5. Textural Properties

The textural properties of the FRNs are shown in Table 5. MLP decreased the hardness of the uncooked FRNs compared to F0. Statistically, the 2 and 4% substitutions did not significantly (*p* > 0.05) affect the hardness, whereas the 6–8% substituted samples were significantly (*p* < 0.05) different when compared to the control sample. However, the hardness of the noodles with higher MLP substitutions (6 to 8%) was not significantly different. As confirmed in the SEM analysis, the hardness values decreased due to an increase in the minor cracks in the noodles. Similar results of a decrease in the hardness of pasta were observed by Szydłowska-Tutaj et al. [14].

The TPA of the cooked noodles showed an insignificant (*p* > 0.05) effect of MLP on hardness, adhesiveness, gumminess, chewiness, and cohesiveness compared to the F0 noodles. The F8 sample had significantly lower gumminess and cohesiveness values compared to the F2 sample. As observed in the SEM analysis, although there were minor cracks, they were filled during cooking due to starch swelling and gelatinization, which resulted in no significant difference in the textural properties of the cooked noodles. In particular, MLP decreased the hardness of the uncooked noodles, whereas it insignificantly impacted all the texture properties of the cooked noodles. 

### 3.6. Total Phenolic Content (TPC), Antioxidant Capacity, and Total Flavonoid Content (TFC)

The results presented in Table 6 indicate that MLP had a high TPC, AOX capacity, and TFC, which were found to be higher than the values reported in a previous study by Assefa et al. [35]. Incorporating MLP into the noodles resulted in a significant (*p* < 0.05) increase in the TPC, AOX capacity, and TFC. Interestingly, there was no significant (*p* > 0.05) change in the AOX capacity between the F2-F4, F4-F6, and F6-F8 samples. Notably, the F0 sample had a higher TPC, AOX capacity, and TFC than the RF, indicating the positive effect of extrusion on increasing the bioactive compounds. However, compared to the formulations, the fortified noodles had lower values of the TPC, AOX capacity, and TFC, reflecting a reduction in the bioactive compounds of marjoram through extrusion. This may be due to the release of phenolic compounds and flavonoids in free form during extrusion, particularly for the F0 noodles. Contrariwise, for the FRNs, these free-form phenolics were reduced through extrusion, possibly due to the high-temperature conditions. Similar findings were reported by Zhang et al. [36].

### 3.7. FTIR Analysis

**Figure 7:**
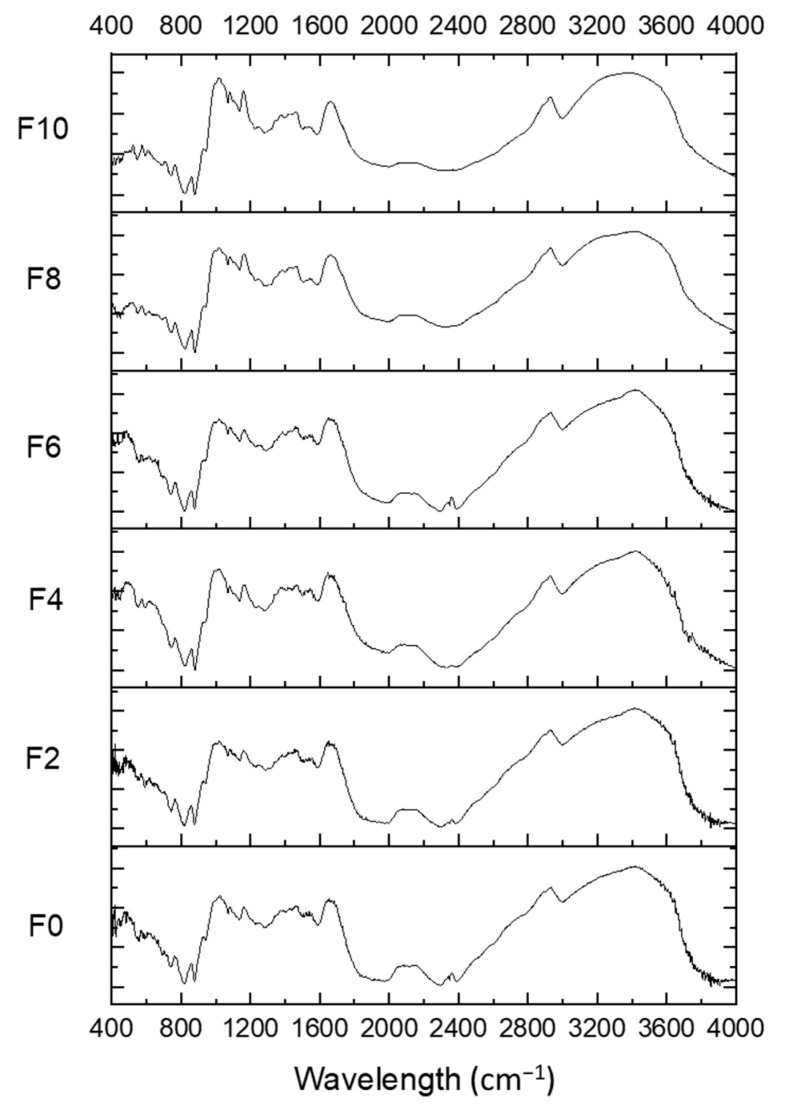
FTIR spectra of F0 and FRN samples. F0 represents 0% MLP, F2 represents 2% MLP, F4 represents 4% MLP, F6 represents 6% MLP, F8 represents 8% MLP, and F10 represents 10% MLP.

The FTIR spectra of all the formulations were plotted against the wavenumber (400–4000 cm^−1^) to evaluate the interaction of MLP with the RF (Figure 7). The FTIR spectra of all the FRNs were similar to the FTIR spectra of F0. There were no alterations in the chemical bonds; only physical modifications due to the extrusion process were observed. The absorbance bands at 995, 1020, and 1047 cm^−1^ are very sensitive and represent the ordered and crystalline structures of starches [29]. Slight changes in the peak intensities in the 990–1050 cm^−1^ region due to the extrusion process were noticed. The short-range molecular order and amorphous region were characterized by the absorbance ratios R1047/1022 and R1022/995, respectively (Table 7).

Compared with the RF, all the noodles had higher R1047/1022 ratios and lower R1022/995 ratios, indicating the presence of a higher short-range molecular order. Marjoram fortification significantly decreased the R1022/995 ratio, while it increased the R1047/1022 ratio. It can be observed that R1047/1022 increased with the F6 formulation and then decreased, indicating that the excessive addition of marjoram prohibits the formation of a short-range order structure. Lu et al. [37] validated similar trends when rice peptides were added to rice starch, Geng et al. [38] validated them when supplementing rice noodles with vetch starch, and Yadav et al. [29] upheld the results for instant low-glycemic-index rice.

### 3.8. XRD Analysis

**Figure 8:**
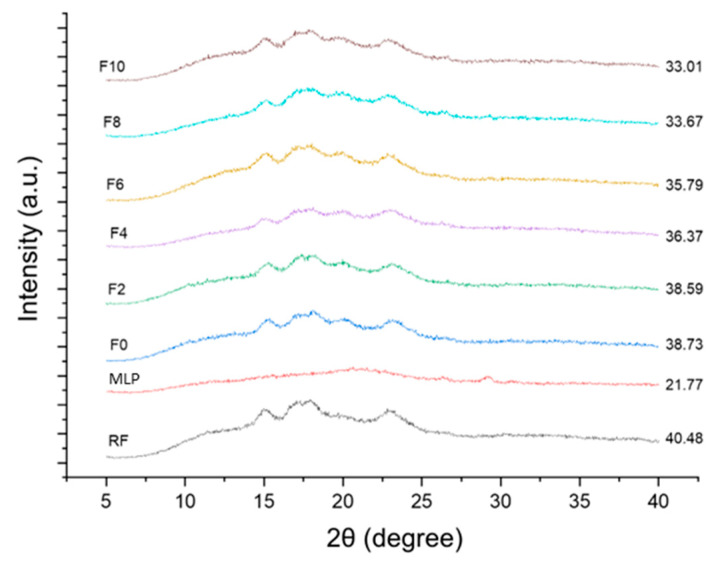
X-ray diffraction pattern of RF, MLP, F0, and FRN samples. RF represents rice flour, MLP represents marjoram leaf powder, F0 represents 0% MLP, F2 represents 2% MLP, F4 represents 4% MLP, F6 represents 6% MLP, F8 represents 8% MLP, and F10 represents 10% MLP.

The XRD analysis of the RF, MLP, and the FRN samples is shown in Figure 8. The crystallinity of the RF was highest (40.48%), and that of MLP was lowest (21.77%). The extrusion caused a reduction in the crystallinity of all the noodle samples. The addition of MLP decreased the crystallinity from 38.59% (F2) to 33.01% (F10). All the extruded samples showed crystallinity patterns (A-type patterns) similar to those of the RF, i.e., characteristic peaks at 15°, 17°, 18°, and 23°. Due to extrusion, V-type crystallinity was evident in each extruded sample at 2θ of 20°. The results found crystalline structures in the FRNs after extrusion, which was also shown through the FTIR analysis. A loss of crystallinity in the extruded product was mainly associated with gelatinization and amylose–lipid complex formation. A similar loss in crystallinity was observed by Yadav et al. [29]. This decline in crystallinity led to the loosening of the rigid structure of the noodles as evidenced by the rise in the cracks shown in the SEM images (Figure 5). This phenomenon resulted in a decrease in CT and an improvement in the firmness of the FRNs. Bhandari et al. [15] discovered a similar correlation between the reduction in the CT of pasta and the decrease in crystallinity due to the addition of herbs ranging from 2.5% to 10%.

### 3.9. Sensory Properties

The effect of the MLP addition on the sensory properties of the noodles is shown in Table 8. The increase in the MLP levels resulted in a decline in the rating of the color parameters of the noodles. However, no significant (*p* > 0.05) effect was observed for up to 4% MLP, and the lowest color rating was obtained by F10. Compared to F0, F2 and F4 showed no significant difference (*p* > 0.05) in almost all the sensory attributes. The MLP addition resulted in a significant improvement in the aroma. A further increase in the MLP levels caused a decrease in the aroma rating, possibly due to its rugged aroma. A significant (*p* < 0.05) decrease in the taste and appearance of the noodles was observed after a 4% addition of MLP. The F2 samples obtained the highest overall acceptability followed by F0 and F4, while the further addition of MLP resulted in a significant (*p* < 0.05) decrease in the overall acceptability of the noodles. In terms of sensory acceptability, the panelist gave the highest rating to the F2 and F4 FRNs with no significant (*p* > 0.05) difference between them. However, the sensory results showed a significant (*p* < 0.05) decrease in the sensory properties at higher levels of marjoram. These results are in accordance with the texture, cooking, rheological, and physicochemical studies. Fortification with moringa in wheat bread by Govender and Siwela [39] substantiated similar results regarding sensory properties.

### 3.10. Principal Component Analysis

**Figure 9:**
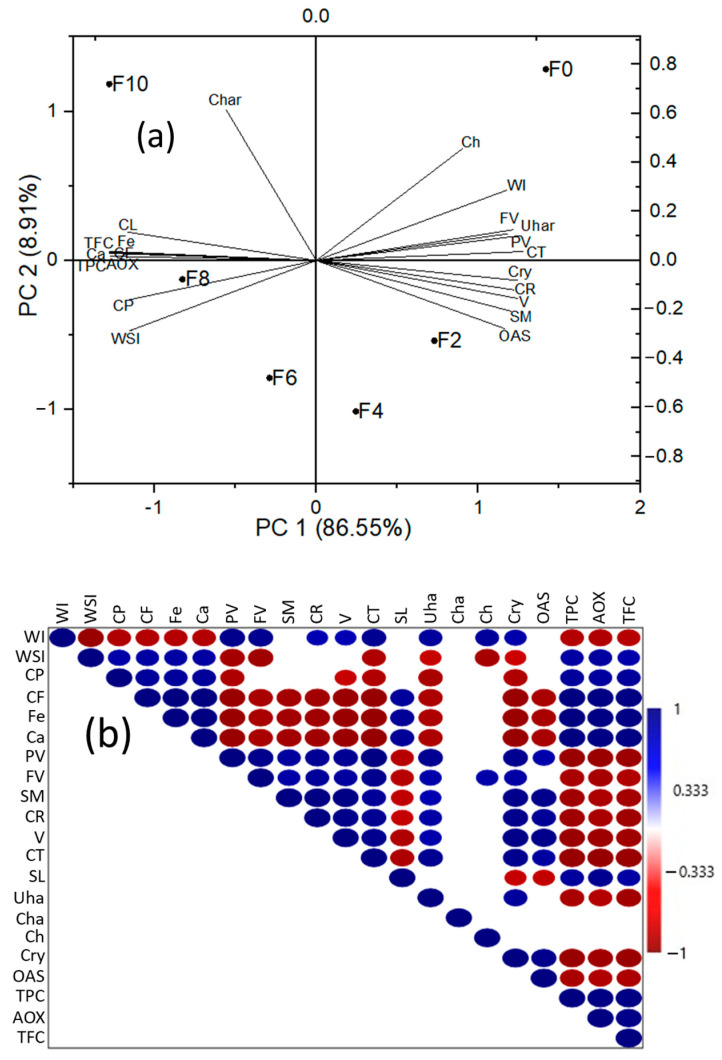
Correlation analysis: (**a**) biplot of PCA of FRNs and (**b**) Pearson correlation analysis. WI represents whiteness index, WSI represents water solubility index, CP represents crude protein, CF represents crude fiber, Fe represents iron, Ca represents calcium, PV represents peak viscosity, FV represents final viscosity, SM represents storage modulus at 43.7 rad/s, CR represents creep at 274.211 s, V represents viscosity at 0.398 s^−1^, CT represents cooking time, SL represents solid loss, Uha represents uncooked noodle hardness, Cha represents cooked noodle hardness, Ch represents chewiness of cooked noodles, Cry represents crystallinity %, OAS represents overall acceptability of noodles, TPC represents total phenolic content, AOX represents antioxidant capacity, and TFC represents total flavonoid content. The colored ellipse in the upper triangle showed the correlation significance at *p* < 0.05. The blue color shows a positive correlation, and the maroon color shows a negative correlation. F0 represents 0% MLP, F2 represents 2% MLP, F4 represents 4% MLP, F6 represents 6% MLP, F8 represents 8% MLP, and F10 represents 10% MLP.

The PCA was applied to locate the appropriate MLP addition levels for the noodles and the correlations among related noodle properties. The WI, WSI, protein, fiber, iron, calcium, PV, FV, gel rheological properties, CT, SL, hardness of the cooked and uncooked noodles, chewiness of the cooked noodles, crystallinity percentage, overall acceptability, TPC, AOX capacity, and TFC were used in the PCA. The biplot of the PCA is shown in Figure 9a. PC1 and PC2 held 95.46% of the data and had eigenvalues greater than one. The F2 and F4 samples were positioned close to each other, showing similar properties. F0 and F10 were quite distant and were in two opposite quadrants, reflecting disparate properties from other samples and between each other. The F6 and F8 samples were also close to each other but were not as close as F2 and F4. Most of the noodles’ properties were shown by PC1, except the texture of the cooked noodles, which was shown by PC2. The PCA biplot revealed a positive correlation among the nutritional properties (protein, fiber, calcium, and iron), physicochemical properties (water solubility index), TPC, AOX capacity, and TFC of the FRNs. On the other hand, a negative correlation was observed between the color properties, such as the whiteness index, and both the nutritional and physicochemical properties. A similar positive correlation was seen among the pasting, cooking, uncooked noodle hardness, rheological, and sensory properties. However, a negative correlation between the cooking, color, and physicochemical properties and the pasting, cooking, rheological, and sensory properties could also be seen. Figure 9b describes the Pearson correlation analysis and shows results similar to the PCA. However, no significant (*p* > 0.05) correlation was obtained between the rheological properties and crude protein. The cooked noodle texture had no significant (*p* > 0.05) correlation with the nutritional, rheological, cooking, and uncooked texture properties. The sensory properties also had no significant (*p* > 0.05) correlation with the color, physicochemical, and noodle textural properties.

## 4. Conclusions

Fortification with MLP enhanced the iron, calcium, protein, and fiber of the noodles with an improvement in the cooking properties and little effect on the textural properties. MLP has a high content of bioactive compounds, which resulted in an increase in the TPC, AOX capacity, and TFC of the noodles. Furthermore, there was no change in the bonds, but the crystallinity percentage reduced. The color of the noodles changed significantly with the addition of natural fortificants but was moderately linked to up to 4% MLP by the sensory panelist. The rheological study showed the minimal effect of fortification on the gelling strength of the fortified noodles. Overall, the study showed an improvement in the nutritional properties due to the addition of MLP; however, better textural, sensory, cooking, and rheological properties were observed at lower FLs (up to 4% MLP).

## Figures and Tables

**Table 1:** Nutritional composition of RF, MLP, F0, and FRNs with different levels of MLP addition.

Property	Moisture (g/100 g)	Crude Protein (g/100 g)	Crude Fiber (g/100 g)	Crude Fat (g/100 g)	Ash (g/100 g)	Carbohydrate (g/100 g)	Fe (mg/100 g)	Zn (mg/100 g)	Ca (mg/100 g)
RF	12.08 ^A^ ± 0.13	7.00 ^C^ ± 0.07	0.45 ^G^ ± 0.06	0.06 ^F^ ± 0.01	0.34 ^G^ ± 0.06	80.08 ^C^ ± 0.08	0.22 ^G^ ± 0.03	1.19 ^B^ ± 0.12	6.00 ^G^ ± 0.10
MLP	10.52 ^D^ ± 0.17	9.72 ^A^ ± 0.03	16.15 ^A^ ± 0.03	2.92 ^A^ ± 0.03	12.05 ^A^ ± 0.03	48.63 ^G^ ± 0.07	53.32 ^A^ ± 0.13	3.49 ^A^ ± 0.30	200.00 ^A^ ± 0.50
F0	11.08 ^cC^ ± 0.23	6.37 ^bD^ ± 0.11	0.41 ^fG^ ± 0.05	0.05 ^eF^ ± 0.03	0.31 ^fG^ ± 0.05	81.78 ^aA^ ± 0.06	0.21 ^fG^ ± 0.02	1.13 ^dB^ ± 0.05	5.70 ^fG^ ± 0.05
F2	11.61 ^abAB^ ± 0.06	6.44 ^bD^ ± 0.07	0.72 ^eF^ ± 0.06	0.11 ^deEF^ ± 0.03	0.54 ^eF^ ± 0.06	80.58 ^bB^ ± 0.17	1.27 ^eF^ ± 0.01	1.18 ^cdB^ ± 0.06	9.59 ^eF^ ± 0.06
F4	11.37 ^bcBC^ ± 0.17	7.11 ^aBC^ ± 0.03	1.08 ^dE^ ± 0.06	0.17 ^cdDE^ ± 0.02	0.81 ^dE^ ± 0.06	79.47 ^cD^ ± 0.03	2.34 ^dE^ ± 0.01	1.28 ^bcB^ ± 0.06	13.76 ^dE^ ± 0.06
F6	11.37 ^bcBC^ ± 0.26	7.16 ^aBC^ ± 0.05	1.39 ^cD^ ± 0.03	0.23 ^bcCD^ ± 0.03	1.04 ^cD^ ± 0.03	78.81 ^dE^ ± 0.15	3.41 ^cD^ ± 0.03	1.33 ^abB^ ± 0.03	17.64 ^cD^ ± 0.03
F8	11.95 ^aA^ ± 0.08	7.22 ^aB^ ± 0.02	1.71 ^bC^ ± 0.02	0.28 ^abBC^ ± 0.03	1.27 ^bC^ ± 0.02	77.57 ^eF^ ± 0.01	4.47 ^bC^ ± 0.01	1.37 ^abB^ ± 0.02	21.52 ^bC^ ± 0.02
F10	11.26 ^bcBC^ ± 0.18	7.27 ^aB^ ± 0.03	2.02 ^aB^ ± 0.03	0.34 ^aB^ ± 0.03	1.51 ^aB^ ± 0.03	77.60 ^eF^ ± 0.06	5.53 ^aB^ ± 0.01	1.42 ^aB^ ± 0.03	25.40 ^aB^ ± 0.03

All the values are mean ± SD. Values with different lowercase letters in the same column differ significantly (*p* < 0.05) among F0 to F10 samples. Values with different capital letters in the same column differ significantly (*p* < 0.05) among all samples. RF represents rice flour, MLP represents marjoram leaf powder, F0 represents 0% MLP, F2 represents 2% MLP, F4 represents 4% MLP, F6 represents 6% MLP, F8 represents 8% MLP, and F10 represents 10% MLP.

**Table 2:** Color characteristics of the RF, MLP, and FRN.

Sample	L	a	b	ΔE	WI
RF	96.69 ^A^ ± 0.08	−0.22 ^F^ ± 0.04	5.55 ^E^ ± 0.10	-	93.53 ^A^ ± 0.11
MLP	49.00 ^F^ ± 0.61	2.00 ^D^ ± 0.07	25.65 ^A^ ± 0.54	-	42.88 ^F^ ± 0.31
F0	80.16 ^aB^ ± 0.82	0.22 ^dE^ ± 0.07	14.16 ^cD^ ± 0.52	-	75.63 ^aB^ ± 1.15
F2	64.93 ^bC^ ± 0.31	3.01 ^cC^ ± 0.07	22.91 ^aB^ ± 0.29	17.79 ^d^ ± 0.51	58.00 ^bC^ ± 0.52
F4	57.26 ^cD^ ± 0.04	3.91 ^bB^ ± 0.01	23.67 ^aB^ ± 0.10	25.07 ^c^ ± 0.07	50.99 ^cD^ ± 0.08
F6	51.52 ^dE^ ± 0.46	3.91 ^bB^ ± 0.01	22.99 ^aB^ ± 0.22	30.21 ^b^ ± 0.48	46.20 ^dE^ ± 0.42
F8	48.52 ^eF^ ± 0.36	4.87 ^aA^ ± 0.07	21.71 ^bC^ ± 0.08	33.40 ^a^ ± 0.42	43.40 ^eF^ ± 0.39
F10	48.48 ^eF^ ± 0.06	4.71 ^aA^ ± 0.02	21.67 ^bC^ ± 0.03	32.87 ^a^ ± 0.08	43.91 ^eF^ ± 0.09

All the values are mean ± SD. Values with different lowercase letters in the same column differ significantly (*p* < 0.05) among F0 to F10 samples. Values with different capital letters in the same column differ significantly (*p* < 0.05) among all samples. ΔE and WI are the overall color difference and whiteness index, respectively. L, a, and b are the color parameters of samples. RF represents rice flour, MLP represents marjoram leaf powder, F0 represents 0% MLP, F2 represents 2% MLP, F4 represents 4% MLP, F6 represents 6% MLP, F8 represents 8% MLP, and F10 represents 10% MLP.

**Table 3:** Water absorption and water solubility indices of raw ingredients, F0, and FRNs.

Sample	WAI (g/g)	WSI (%)
RF	2.43 ^C^ ± 0.01	2.02 ^CD^ ± 0.49
MLP	4.55 ^A^ ± 0.02	25.51 ^A^ ± 0.94
F0	3.11 ^aB^ ± 0.22	1.25 ^dB^ ± 0.11
F2	2.97 ^aB^ ± 0.24	3.02 ^cBC^ ± 0.08
F4	3.01 ^aB^ ± 0.08	3.40 ^bB^ ± 0.11
F6	2.92 ^aB^ ± 0.26	3.51 ^bB^ ± 0.15
F8	2.93 ^aB^ ± 0.05	3.95 ^aB^ ± 0.01
F10	2.89 ^aB^ ± 0.34	4.00 ^aB^ ± 0.13

All the values are mean ± SD. Values with different lowercase letters in the same column differ significantly (*p* < 0.05) among F0 to F10 samples. Values with different capital letters in the same column differ significantly (*p* < 0.05) among all samples. WAI represents water absorption index, WSI represents water solubility index, RF represents rice flour, MLP represents marjoram leaf powder, F0 represents 0% MLP, F2 represents 2% MLP, F4 represents 4% MLP, F6 represents 6% MLP, F8 represents 8% MLP, and F10 represents 10% MLP.

**Table 4:** Pasting properties of RF, F0, and FRN samples.

Sample	Peak Temp (°C)	Peak Viscosity (Pas)	Holding Strength (Pas)	Breakdown (Pas)	Final Viscosity (Pas)	Setback from Peak (Pas)	Setback from Trough (Pas)
RF	95.30 ^A^ ± 0	2187.00 ^A^ ± 41.01	1453.00 ^A^ ± 18.38	734.00 ^A^ ± 22.34	3164.00 ^A^ ± 80.61	976.40 ^C^ ± 39.03	2430.00 ^A^ ± 57.98
F0	95.15 ^aB^ ± 0.07	831.75 ^aB^ ± 70.22	555.95 ^aB^ ± 21.14	275.85 ^aB^ ± 91.29	1415.50 ^aB^ ± 74.25	583.25 ^bB^ ± 4.03	1139.00 ^aB^ ± 16.97
F2	95.10 ^aB^ ± 0	705.90 ^abBC^ ± 4.81	516.75 ^abBC^ ± 4.60	189.15 ^abBC^ ± 0.21	1194.00 ^bcCD^ ± 5.66	488.40 ^aA^ ± 0.99	1005.00 ^bcBC^ ± 5.66
F4	95.10 ^aB^ ± 0	642.90 ^bcCD^ ± 24.75	515.75 ^abBC^ ± 10.54	127.15 ^abC^ ± 14.35	1220.00 ^bC^ ± 5.66	577.25 ^bAB^ ± 18.88	1093.00 ^abBC^ ± 8.49
F6	95.10 ^aB^ ± 0	563.45 ^cdDE^ ± 4.17	470.15 ^bcCD^ ± 1.77	93.35 ^bC^ ± 5.9	1147.00 ^bcdCD^ ± 8.49	583.70 ^bB^ ± 12.73	1054.00 ^abcBC^ ± 14.14
F8	95.20 ^aAB^ ± 0	509.95 ^cdDE^ ± 12.37	422.50 ^cD^ ± 20.51	87.41 ^bC^ ± 8.19	1055.00 ^cdCD^ ± 55.15	545.15 ^abAB^ ± 42.36	967.60 ^cC^ ± 62.79
F10	95.10 ^aB^ ± 0	477.50 ^dE^ ± 33.66	408.00 ^cD^ ± 23.48	69.50 ^bC^ ± 10.25	1030.00 ^dD^ ± 22.63	552.85 ^abAB^ ± 10.96	960.80 ^cC^ ± 12.45

All the values are mean ± SD. Values with different lowercase letters in the same column differ significantly (*p* < 0.05) among F0 to F10 samples. Values with different capital letters in the same column differ significantly (*p* < 0.05) among all samples. RF represents rice flour, F0 represents 0% MLP, F2 represents 2% MLP, F4 represents 4% MLP, F6 represents 6% MLP, F8 represents 8% MLP, and F10 represents 10% MLP.

**Table 5:** Textural properties of uncooked and cooked noodles based on F0 and FRNs.

Sample	Uncooked	Cooked				
	Hardness (N)	Hardness (N)	Adhesiveness (J × 10^−3^)	Gumminess (N)	Chewiness (J × 10^−3^)	Cohesiveness
F0	117.04 ^a^ ± 9.86	17.41 ^ab^ ± 0.47	0.43 ^a^ ± 0.15	10.65 ^ab^ ± 1.94	204.93 ^a^ ± 55.01	0.61 ^ab^ ± 0.10
F2	111.26 ^ab^ ± 16.17	16.80 ^b^ ± 0.39	0.27 ^a^ ± 0.12	12.40 ^a^ ± 1.12	10.63 ^b^ ± 1.19	0.74 ^a^ ± 0.06
F4	94.31 ^b^ ± 12.54	15.87 ^b^ ± 0.22	0.13 ^a^ ± 0.06	10.54 ^ab^ ± 0.11	8.63 ^b^ ± 0.55	0.66 ^ab^ ± 0.01
F6	66.96 ^c^ ± 15.58	16.31 ^b^ ± 0.58	0.27 ^a^ ± 0.06	9.89 ^ab^ ± 0.42	8.10 ^b^ ± 0.30	0.60 _ab_ ± 0.04
F8	68.13 ^c^ ± 6.51	17.03 ^b^ ± 0.68	0.43 ^a^ ± 0.21	8.63 ^b^ ± 0.62	6.43 ^b^ ± 0.49	0.51 ^b^ ± 0.06
F10	70.58 ^c^ ± 12.59	19.30 ^a^ ± 1.34	0.20 ^a^ ± 0.17	9.97 ^ab^ ± 0.75	7.53 ^b^ ± 0.46	0.52 ^b^ ± 0.03

All the values are mean ± SD, and different superscripts in the same column show significant differences (*p* < 0.05). F0 represents 0% MLP, F2 represents 2% MLP, F4 represents 4% MLP, F6 represents 6% MLP, F8 represents 8% MLP, and F10 represents 10% MLP.

**Table 6:** Contents of TPC, AOX capacity, and TFC of raw ingredients, F0, and FRN samples.

Sample	TPC (mg GAE/g DW)	AOX (mg GAE/g DW)	TFC (mg QE/g DW)
RF	2.1242 ^B^ ± 0.1717	0.02010 ^B^ ± 0.0006	0.0949 ^B^ ± 0.0712
MLP	350.8222 ^A^ ± 39.6955	113.0690 ^A^ ± 0.9616	10.0362 ^A^ ± 0.6436
F0	2.2340 ^fB^ ± 0.0174	0.0484 ^eB^ ± 0.0044	0.1068 ^fB^ ± 0.0119
F2	5.4715 ^eB^ ± 0.1502	0.0570 ^dB^ ± 0.0013	0.1632 ^eB^ ± 0.003
F4	9.1300 ^dB^ ± 0.0322	0.0630 ^cdB^ ± 0.0027	0.2492 ^dB^ ± 0.0059
F6	12.6489 ^cB^ ± 0.0107	0.0689 ^bcB^ ± 0.0013	0.3842 ^cB^ ± 0.0034
F8	16.4146 ^bB^ ± 0.2789	0.0727 ^bB^ ± 0.0015	0.4606 ^bB^ ± 0.0066
F10	19.1511 ^aB^ ± 0.0561	0.0823 ^aB^ ± 0.0024	0.5100 ^aB^ ± 0.0017

All the values are mean ± SD. Values with different lowercase letters in the same column differ significantly (*p* < 0.05) among F0 to F10 samples. Values with different capital letters in the same column differ significantly (*p* < 0.05) among all samples. GAE represents gallic acid equivalent, AOX represents antioxidant capacity, TFC represents total flavonoid content, DW represents dry weight. RF represents rice flour, MLP represents marjoram leaf powder, F0 represents 0% MLP, F2 represents 2% MLP, F4 represents 4% MLP, F6 represents 6% MLP, F8 represents 8% MLP, and F10 represents 10% MLP.

**Table 7:** Absorbance ratio of RF, MLP, F0, and FRNs.

Sample	Absorbance			Absorbance Ratio	
	A995	A1022	A1047	R1047/1022	R1022/995
RF	0.411	0.435	0.385	0.88	1.058
MLP	0.123	0.157	0.158	1.01	1.276
F0	0.098	0.103	0.097	0.94	1.050
F2	0.106	0.108	0.103	0.96	1.022
F4	0.070	0.071	0.065	0.92	1.013
F6	0.089	0.090	0.088	0.98	1.016
F8	0.148	0.152	0.144	0.95	1.029
F10	0.410	0.431	0.401	0.93	1.050

RF represents rice flour, MLP represents marjoram leaf powder, F0 represents 0% MLP, F2 represents 2% MLP, F4 represents 4% MLP, F6 represents 6% MLP, F8 represents 8% MLP, and F10 represents 10% MLP.

**Table 8:** Sensory acceptability of F0 and FRNs.

Sample	Color	Appearance	Stickiness	Aroma	Taste	Texture	Overall Acceptability
F0	8.00 ^a^ ± 0.89	6.18 ^abc^ ± 1.33	6.18 ^ab^ ± 1.54	5.36 ^c^ ± 0.81	7.09 ^a^ ± 0.83	6.64 ^a^ ± 1.69	7.00 ^ab^ ± 1.25
F2	7.64 ^a^ ± 1.03	7.82 ^a^ ± 1.47	7.36 ^a^ ± 1.43	6.73 ^ab^ ± 0.90	6.64 ^ab^ ± 0.81	7.45 ^a^ ± 0.82	7.82 ^a^ ± 0.75
F4	7.64 ^a^ ± 0.81	7.00 ^ab^ ± 1.84	7.27 ^a^ ± 0.79	7.27 ^a^ ± 0.79	6.00 ^ab^ ± 0.63	6.36 ^ab^ ± 0.81	6.45 ^b^ ± 0.52
F6	5.45 ^b^ ± 1.86	5.27 ^bcd^ ± 2.05	6.09 ^ab^ ± 1.14	6.91 ^ab^ ± 0.83	5.82 ^b^ ± 0.75	6.00 ^ab^ ± 1.41	5.82 ^b^ ± 0.40
F8	4.73 ^bc^ ± 2.10	4.45 ^cd^ ± 2.58	5.45 ^b^ ± 1.37	6.00 ^bc^ ± 0.63	5.45 ^bc^ ± 1.13	5.09 ^b^ ± 1.22	4.55 ^c^ ± 0.82
F10	3.36 ^c^ ± 2.06	3.73 ^d^ ± 2.05	4.82 ^b^ ± 1.17	5.82 ^bc^ ± 1.40	4.45 ^c^ ± 1.44	5.00 ^b^ ± 0.80	3.09 ^d^ ± 1.45

All values are expressed as mean ± SD. Different superscripts in the same column show significant differences (*p* < 0.05). F0 represents 0% MLP, F2 represents 2% MLP, F4 represents 4% MLP, F6 represents 6% MLP, F8 represents 8% MLP, and F10 represents 10% MLP.

## Data Availability

Data supporting the reported results are available upon request.
